# Efficient vitrification of mouse embryos using the Kitasato Vitrification System as a novel vitrification device

**DOI:** 10.1186/s12958-017-0249-2

**Published:** 2017-04-24

**Authors:** Kenji Momozawa, Atsushi Matsuzawa, Yukio Tokunaga, Shiori Abe, Yumi Koyanagi, Miho Kurita, Marina Nakano, Takao Miyake

**Affiliations:** 1 School of Veterinary Medicine, Kitasato University, Aomori, 034-8628 Japan; 2 Kyoto R&D Laboratory, Mitsubishi Paper Mills Limited, Kyoto, 617-8666 Japan; 3 Miyake Women’s Clinic, Chiba, 266-0032 Japan

**Keywords:** Vitrification, KVS, Embryos, Ultra-rapid cooling, Cryopreservation

## Abstract

**Background:**

Currently, the cryopreservation of embryos and oocytes is essential for assisted reproductive technology (ART) laboratories worldwide. This study aimed to evaluate the efficacy of the Kitasato Vitrification System (KVS) as a vitrification device for the cryopreservation of mouse embryos to determine whether this novel device can be adapted to the field of ART.

**Methods:**

In Experiment 1, blastocysts were vitrified using the KVS. Vitrified blastocysts were warmed and subsequently cultured for 72 h. In Experiment 2, 2-cell-stage embryos were vitrified using the KVS, and vitrified embryos were warmed and subsequently cultured for 96 h. In Experiment 3, we evaluated the in vivo developmental potential of vitrified 2-cell-stage embryos using the KVS, and in Experiment 4, we evaluated the cooling and warming rates for these devices using a numerical simulation.

**Results:**

In Experiment 1, there were no significant differences between the survival rates of the KVS and a control device. However, re-expanded (100%) and hatching (91.8%) rates were significantly higher for blastocysts vitrified using the KVS. In Experiment 2, there were no significant differences between the survival rates, or rates of development to the blastocyst stage, of vitrified and fresh embryos. In Experiment 3, after embryo transfer, 41% of the embryos developed into live offspring. In Experiment 4, the cooling and warming rates of the KVS were 683,000 and 612,000 °C/min, respectively, exceeding those of the control device.

**Conclusions:**

Our study clearly demonstrates that the KVS is a novel vitrification device for the cryopreservation of mouse embryos at the blastocyst and 2-cell stage.

## Background

Currently, the cryopreservation of embryos and oocytes is essential for assisted reproductive technology (ART) laboratories worldwide. To date, several vitrification methods based on ultra-rapid cooling have been developed for the cryopreservation of animal embryos [1–8]. The most important concept of these vitrification methods is to minimize the volume of the extracellular vitrification solution in order to obtain a higher cooling rate. However, to our knowledge, even if the volume of the extracellular solution is decreased, the vitrification solution remains present around the cells. In order to address this issue, we previously developed a novel vitrification system for the cryopreservation of bovine embryos [9]. This simple method vitrifies embryos on a membrane filter, which absorbs excess vitrification solution around the embryos. Accordingly, we believe that this vitrification system can achieve greater and more rapid rates of cooling and warming, while also causing less cryodamage. Additionally, a key feature of this system is that the absorption of excess vitrification solution is easy and stable. However, it is difficult to observe embryos that are deposited on the membrane filter under a stereomicroscope. During the cryopreservation of human embryos, an operator has to observe the embryos on the cryodevice under a stereomicroscope at the time of vitrification. However, we have developed a new vitrification device in which the embryo can easily be observed under a stereomicroscope after it and the vitrification solution are placed on the device absorber. We designated our vitrification system the Kitasato Vitrification System (KVS) and further developed it as a vitrification device for the cryopreservation of embryos. In this study, we examined the efficacy of the KVS in the vitrification of mouse embryos by analyzing the viability of embryos and their development to live offspring after vitrified warming to determine whether this novel device can be adapted to the field of ART. In addition, the direct measurement of cooling and warming for vitrification procedures via a physical set-up has been reported [10, 11]. These previous studies have indicated the importance of the warming rate, rather than the cooling rate, with regard to the survival of mouse oocytes subjected to vitrification. Recently, we developed a measurement system for cooling and warming rates during vitrification procedures through the analysis of thermal transfer using a numerical simulation. Therefore, in this study, we also evaluated the relationship of viability with cooling and warming rates during vitrification procedures using the KVS.

## Methods

### Chemicals

Chemicals were obtained from Sigma-Aldrich (St. Louis, MO, USA), unless otherwise indicated.

### Experimental design

#### Experiment 1

Effect of absorbing excess vitrification solution around embryos on the viability of vitrified-warmed mouse embryos at the blastocyst stage.

Female ICR mice (8–16 weeks old) were induced to superovulate via an intraperitoneal (i.p.) injection of 10 IU of equine chorionic gonadotropin (eCG, ASKA Pharmaceutical, Tokyo, Japan) and 10 IU of human chorionic gonadotropin (hCG, ASKA Pharmaceutical) administered 48 h apart. Fourteen hours after hCG injection, the females were euthanized, and their oviductal ampulla were removed. Cumulus-intact eggs released from the oviductal ampulla were transferred to a drop of TYH [12] medium. The spermatozoa were collected from the cauda epididymides of male mice (18–21 weeks). The collected spermatozoa were submerged into a drop of TYH medium and incubated for 90 min under 5% CO_2_ in humidified air at 37 °C. Sperm suspension preincubated for 90 min was added to the drop of TYH medium containing the cumulus intact eggs. The final concentration of spermatozoa was 2 × 10^5^ cells/mL. After 6 h of gamete coincubation, oocytes having two pronuclei were washed in KSOM/AA [13]; they were then transferred into a drop of KSOM/AA and cultured up to 90 h under a gas-phase of 5% CO_2_, 5% O_2_, and 90% N_2_ with high humidity at 37 °C. Embryos at the blastocyst stage were used for vitrification.

**Fig. 1:**
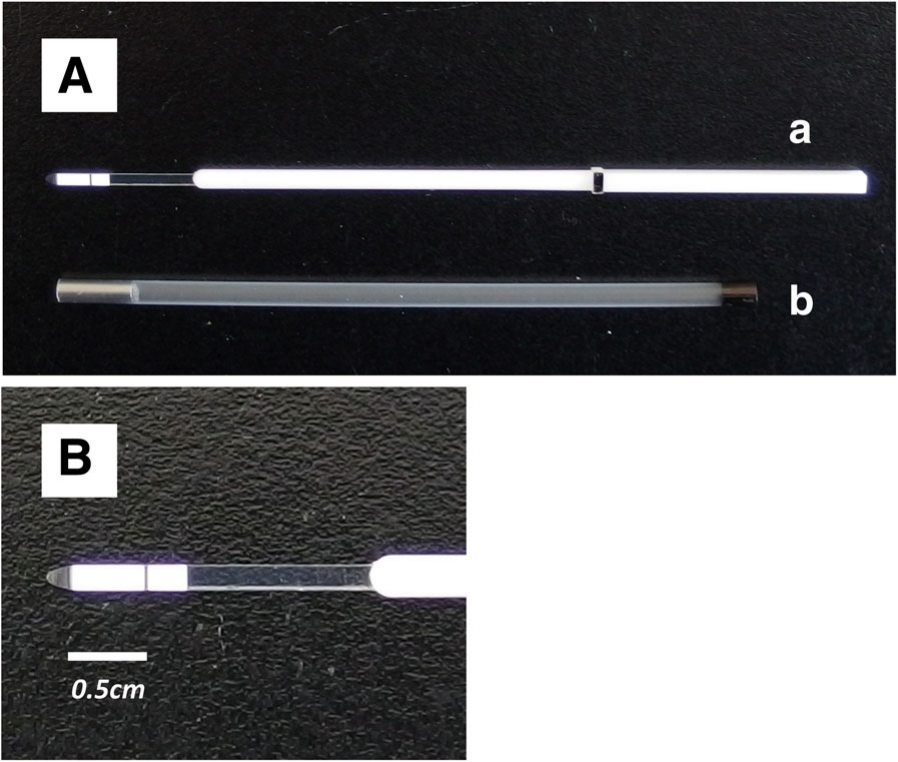
The Kitasato Vitrification System (KVS) for embryo vitrification. **A**: (*a*) The KVS consists of a gripper made of acrylonitrile butadiene styrene resin and a support. (*b*) The protective straw-cap of the device. **B**: The support is composed of polyethylene terephthalate (PET) film and the vitrification solution absorber, which consists of a porous membrane and is placed on the PET film (both ends of the vitrification solution absorber adhered to the PET film). Scale bar = 0.5 cm

**Fig. 2:**
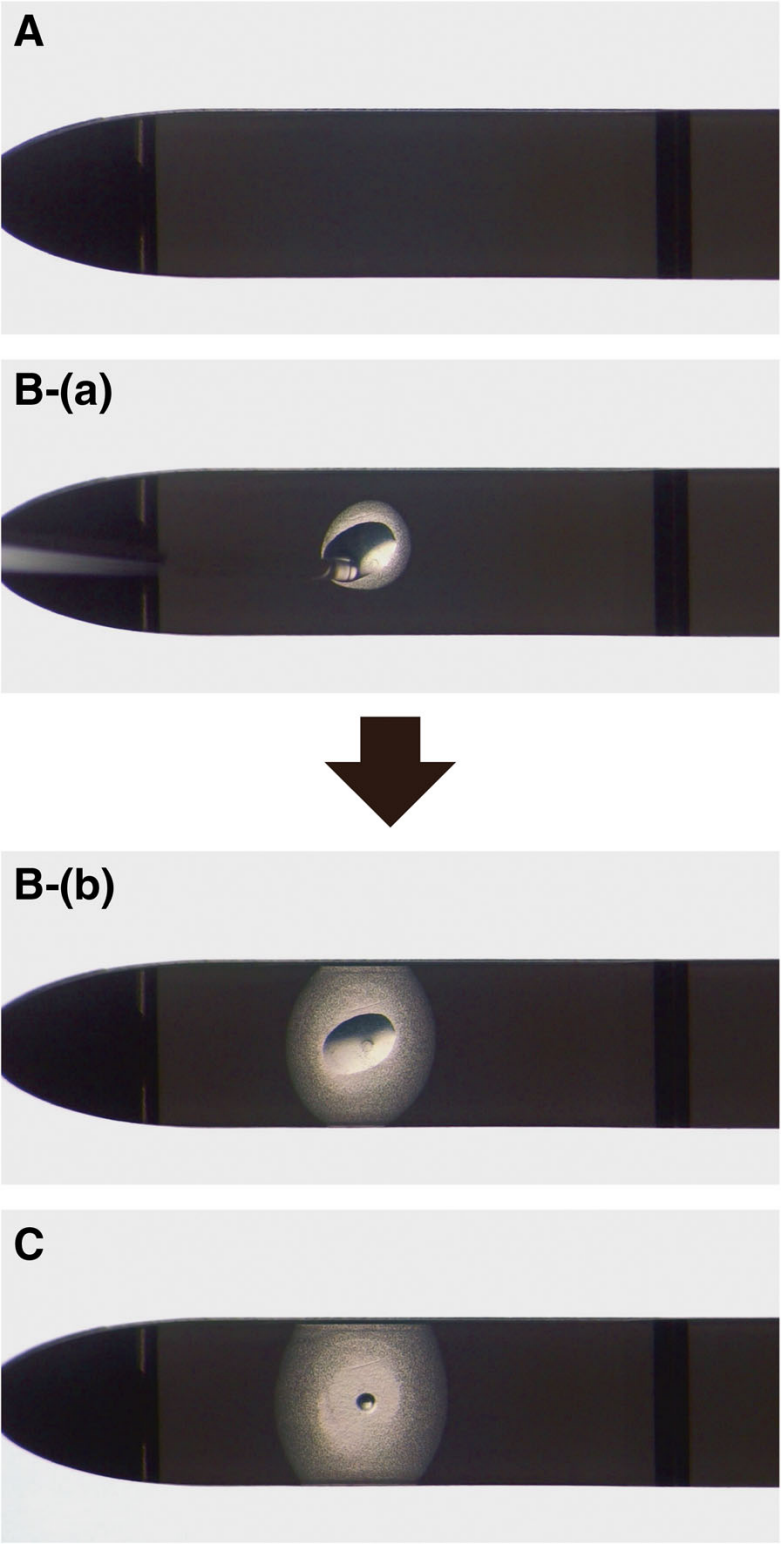
Embryo vitrification procedures using the Kitasato Vitrification System. **A**: The vitrification solution absorber of the device is set on a stereomicroscope. **B**: After an embryo with a small volume (≤0.4 μL) of vitrification solution is placed dropwise on the vitrification absorber (*a*) that embryo can easily be observed under the stereomicroscope (*b*). **C**: Subsequently, the vitrification solution surrounding the embryo is absorbed by the absorber within less than 5–6 s, and the embryo, with a small remaining volume of vitrification solution, can easily be observed

The KVS is composed of a gripper consisting of an acrylonitrile butadiene styrene (ABS) resin, a support, composed of polyethylene terephthalate (PET) film (Fig. 1-A), and a vitrification solution absorber, which consists of a porous membrane that is placed on the PET film (both ends of the vitrification solution absorber adhered to the PET film) (Fig. 1-B). The protocol of this experiment for vitrification and warming was based on the Cryotop safety kit instructions (Kitazato BioPharma, Shizuoka, Japan). Briefly, embryos at the blastocyst stage were equilibrated in equilibration solution for 10 min at room temperature. Subsequently, the embryos were transferred to vitrification solution for 30 s and vitrified using the KVS. After 25–30 s, one embryo with a small amount of vitrification solution (approximately 0.4 μL) was placed on the KVS (Fig. 2-B). Subsequently, the extracellular solution (the vitrification solution covering the embryo) was absorbed by the absorber on the KVS (Fig. 2-C), and the device with the embryo was plunged into liquid nitrogen (LN_2_). Thereafter, the device was inserted into a protective straw-cap in LN_2_. Meanwhile, the PET film without a vitrification solution absorber was used as a control device. The control device is assumed to be one of the other cryodevices used in clinical practice, such as cyroloop [3] and cryotop [5], based on minimum volume cooling. The feature of this control device is that it does not absorb excess vitrification solution around the embryo. The embryo equilibration was conducted as described for the KVS. After equilibration, each embryo with a small amount of vitrification solution (approximately 0.4 μL) was placed on a control device sheet. Subsequently, the control device sheet was plunged into LN_2_. Each cryodevice containing embryos was stored in LN_2_ for 1–14 days.

After storage in LN_2_, the protective straw-cap was removed from the KVS in LN_2_. Thereafter, the KVS with the vitrified embryo was transferred to a thawing solution and warmed at 37 °C. After 1 min, the embryo was transferred to a diluent solution for 3 min at 37 °C. Subsequently, the embryo was transferred to a washing solution for 5 min at 37 °C. Embryos vitrified using the control device were warmed and diluted similarly. Thereafter, the embryos were washed and cultured in culture medium for 72 h under a gas phase of 5% CO_2_, 5% O_2_, and 90% N_2_ with high humidity at 37 °C. After culture for 24 h, development to the re-expanded stage was observed. Embryos that formed the blastocoel were considered viable. Additionally, development to the hatching stage was observed at 24, 48, and 72 h after culture. Experiments were performed six times.

#### Experiment 2

Embryonic development of vitrified-warmed mouse embryos at the 2-cell stage.

Embryo production was performed as described for Experiment 1. At 24 h after insemination, the 2-cell stage embryos were vitrified using the KVS. Vitrification and warming were performed using the same procedure described for Experiment 1. After warming, the embryos were evaluated for viability. Embryos that displayed intact both blastomeres were considered viable. Fresh embryos at the 2-cell stage derived from in vitro production were used as controls. Vitrified and control non-vitrified embryos were cultured for 96 h. After culture, the rate of development to the blastocyst stage was evaluated. Experiments were performed at least three times.

#### Experiment 3

Ability of 2-cell-stage embryos vitrified using the KVS to develop offspring.

In this experiment, vitrified-warmed mouse embryos at the 2-cell stage were used for embryo transfer. Embryos at the 2-cell stage derived from in vitro production were vitrified using the KVS. Vitrification and warming were performed as described for Experiment 1. Fresh embryos at the 2-cell stage derived from in vitro production were used as controls. Eight-week-old ICR mice were used as recipients after being mated with vasectomized males of the same strain. The first day the presence of a vaginal plug was noted was considered day 1 of pseudopregnancy. Vitrified and control non-vitrified embryos were transferred to the oviducts of anesthetized pseudopregnant recipients on day 1. Ten embryos were transferred into each oviduct. The pregnancy and birth rates from each group were recorded and analyzed.

#### Experiment 4

Comparing the cooling and warming rates among different devices using a simulated analysis.

To simulate the vitrification and warming procedures and assess the cooling and warming rates of the KVS and the control device in Experiment 1, we used a thermal transfer-based simulation method. We designed three-dimensional models of embryos supported by the KVS or the control device sheet at each step of the vitrification and warming procedure. The support of the KVS consisted of a PET film with a thin porous membrane as the vitrification solution absorber, with a width and thickness of 1.5 mm and approximately 0.2 mm, respectively. Conversely, the control device sheet, consisting of only PET film, had the same width and thickness as the KVS device sheet. We assessed the temperature at the center of the embryo, which was assumed to be a 0.1-mm cube, with the material properties of water in this simulated experiment. For the KVS, which feature automatic control of the vitrification solution volume, we estimated that the volume of the vitrification solution surrounding the embryo was 1.3 nL based on microscopic images taken prior to immersion in LN_2_ (Fig. 2c). Regarding the control device without a vitrification solution absorber, the vitrification solution volume was 0.4 μL. The material properties for the numerical simulation are presented in Table 1. The thermodynamic properties of each material were considered independent parameters from the temperature, and fixed values at room temperature were used. The material properties of the vitrification and thawing solutions were referenced from a report by Tarakanov et al. [14]. We used ANSYS Fluent (Version 14.5; ANSYS Inc., Canonsburg, PA) for thermo-fluid analysis. For the vitrification procedure, we estimated the temperature at the center of the embryo when the embryo was surrounded by vitrification solution during the transfer from room temperature to LN_2_. For the warming procedure, we estimated the warming rate when the embryo was surrounded by vitrification solution during the transfer from LN_2_ to the thawing solution at 37 °C. Cooling and warming rates were calculated for temperature changes from 20 to −120 °C and from −170 to −20 °C, respectively.

**Table 1:** The properties of the materials used for numerical simulations

Material	Density (kg/m^3^)	Heat capacity (J/kg•K)	Thermal conductivity (W/m•K)
Polyethyleneterephthalate film	1570	1260	0.31
Porous membrane^a^	2170	1050	0.25
Vitrification solution^b^	1255	1657	0.20
Thawing solution^b^	1581	1255	0.28
Embryo^c^	998	4182	0.60

^a^Porous membrane is used as the vitrification solution absorber in the Kitasato Vitrification System

^b^The properties of the vitrification and thawing solution were referenced from a previous report by Tarakanov et al. [14]

^c^The properties of embryos matched those of water at 20 °C

### Statistical analysis

Percentage data on embryo survival, development to blastocysts, hatching blastocysts, and birth rates after embryo transfer were analyzed using one-way ANOVA.

## Results

### Experiment 1

Effect of absorbing excess vitrification solution around embryos on the viability of vitrified-warmed mouse embryos at the blastocyst stage.

The results of Experiment 1 are presented in Table 2. There were no significant differences in the survival rates between the devices. However, the re-expanded blastocyst rates using the KVS were significantly higher than those using the no-absorbing device (100% vs. 93.4%, *P* < 0.05). Furthermore, embryo-hatching rates at 72 h after culture using the KVS were significantly higher than those using the no-absorbing device (91.8% vs. 77.0%, *P* < 0.01).

**Table 2:** Effect of absorbing the extracellular vitrification solution on the viability of vitrified-warmed mouse embryos at the blastocyst stage

Device	Absorbing	No. of embryos examined	No. of embryos survived (%)	No. of embryos re-expanded (%)	No. of hatching embryos (%)
Control device	−	61	61 (100)	57 (93.4)^a^	47 (77.0)^c^
KVS	+	61	61 (100)	61 (100)^b^	56 (91.8)^d^

*KVS* Kitasato Vitrification System

^a,b,c,d^Values in the same column with different superscripts are significantly different (*P* < 0.05 for a vs. b and *P* < 0.01 for c vs. d)

### Experiment 2

Embryonic development of vitrified-warmed mouse embryos at the 2-cell stage.

The results of Experiment 2 are presented in Table 3. There were no significant differences in survival rates between vitrified and fresh embryos. Furthermore, there were no significant differences in the rates of development to the blastocyst stage between vitrified and fresh embryos.

**Table 3:** Embryonic development of vitrified-warmed mouse embryos at the 2-cell stage

Embryos	No. of embryos examined	No. of embryos	
		Survived (%)	Developed to blastocysts on day 4 (%)
Fresh	112	112 (100)	109 (97.3)
Vitrified	100	97 (97.0)	91 (91.0)

### Experiment 3

Ability of 2-cell-stage embryos vitrified using the KVS to develop offspring.

The results of Experiment 3 are presented in Table 4. Embryos at the 2-cell stage derived via in vitro production were transferred to pseudopregnant females. All females that received transferred embryos became pregnant. There were no significant differences in birth rates between vitrified and fresh embryos.

**Table 4:** Term development of 2-cell-stage embryos vitrified using the Kitasato Vitrification System

Embryos	No. of Embryos transferred	No. of recipients	No. of pregnancies (%)	Offspring (%) (Mean ± SEM)
Fresh	100	5	5 (100)	56 (56.0 ± 6.7)
Vitrified	100	5	5 (100)	41 (41.0 ± 4.1)

*SEM* standard error of the mean

### Experiment 4

Comparison of cooling and warming rates between the different devices using a simulated analysis.

**Fig. 3:**
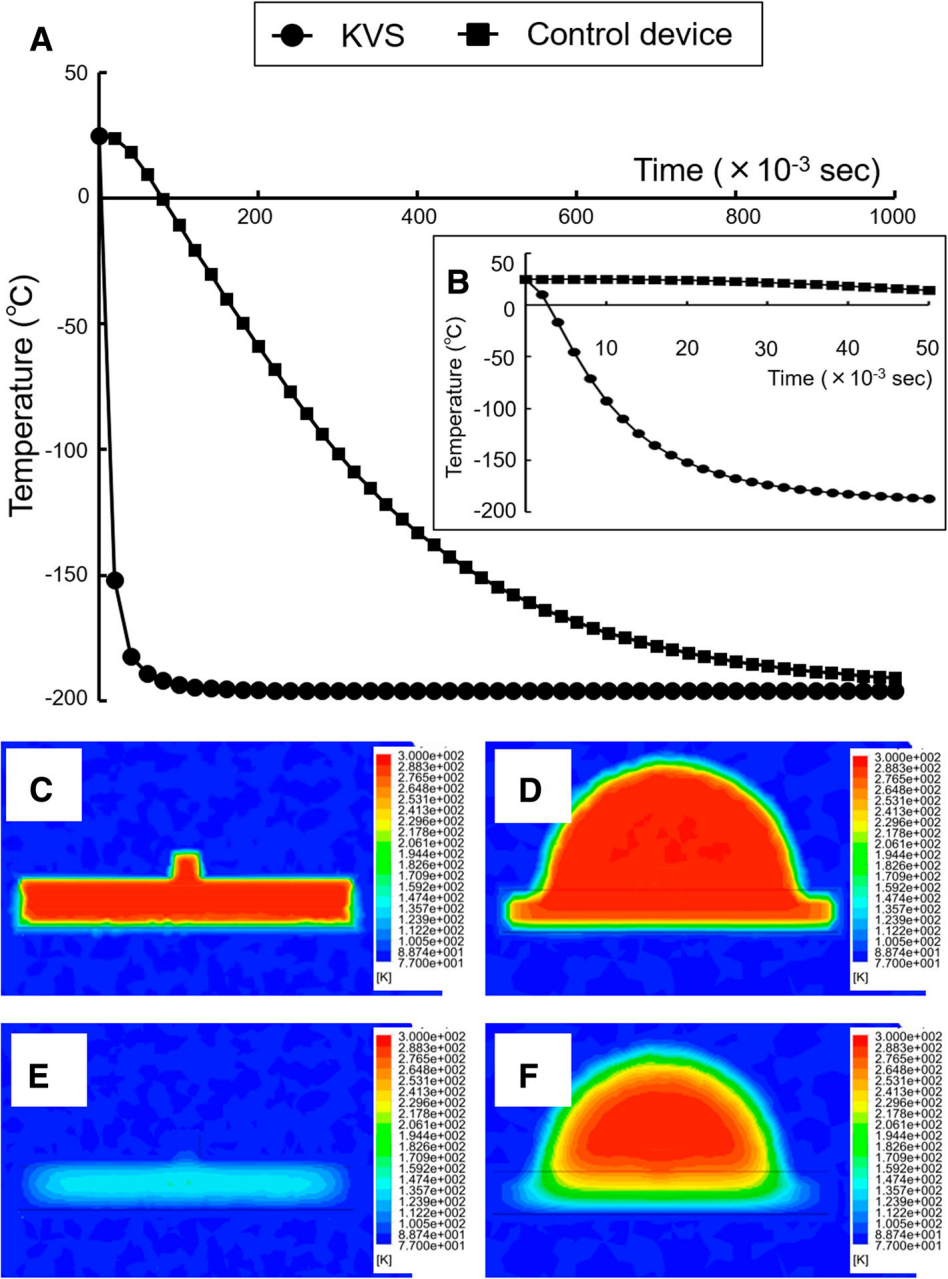
The vitrification dynamics of the Kitasato Vitrification System (KVS) and the Control device. **a**-**b**: The embryo temperature in the vitrification procedure was calculated using a thermal transfer-based simulation method. The temperature in the center of the embryo during the vitrification procedure was plotted using the simulation result for the KVS and the control device. Temperature changes are shown every 0.02 s from liquid nitrogen (LN_2_) immersion for 1 s (**a**) and every 0.002 s from immersion for 0.05 s (**b**). **c**-**f**: Illustration of the thermal distribution of a wide cross-section of embryos on the KVS support (**c** and **e**) or the control device sheet (**d** and **f**) in the vitrification procedure. Thermal distributions immediately after immersion in LN_2_ are shown in (**c** and **d**). The thermal distributions at 0.05 s after immersion are shown in (**e** and **f**)

**Fig. 4:**
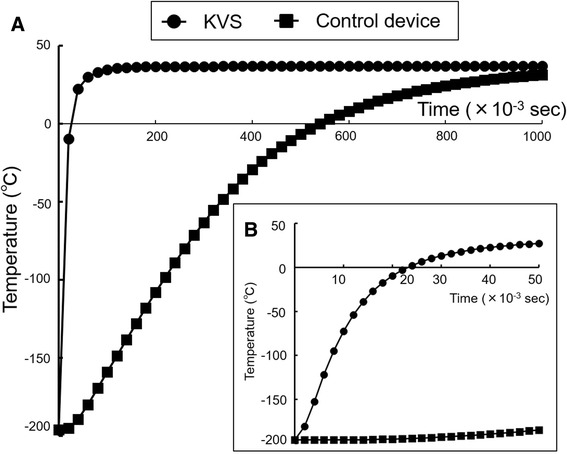
Embryo temperature in the warming procedure was calculated using a thermal transfer-based simulation method. **a**-**b**: The temperature in the center of the embryo during the warming procedure was plotted using the simulation result for the Kitasato Vitrification System and the control device. The temperature changes are shown every 0.02 s from thawing solution immersion for 1 s (**a**) and every 0.002 s from immersion for 0.05 s (**b**)

The vitrification dynamics of both devices estimated with thermal transfer-based simulation methods using numerical modeling are shown in Fig. 3. The embryo temperature in the KVS device was sufficiently cool until approximately 0.05 s after LN_2_ immersion (Fig. 3b). On the other hand, in the control device, it took approximately 1 s to cool down to the same temperature as the external environment (Fig. 3a). An analysis of the thermal distribution during the vitrification procedure is shown in Fig. 3c–f. For the KVS, the embryos on the support were preferentially cooling compared with the support itself (Fig. 3e). On the contrary, the embryos on the control device sheet still had thermal energy at 0.05 s after LN_2_ immersion (Fig. 3f). The dynamics during the warming procedure are shown in Fig. 4. In embryos vitrified using the KVS device, it was estimated that the temperature quickly rose after immersion in the thawing solution (Fig. 4b). On the other hand, in the control device, it took approximately 1 s to warm up (Fig. 4a). The cooling and warming rates are presented in Table 5. Both rates were more rapid for the KVS with the vitrification solution absorber than the control device without the vitrification solution absorber.

**Table 5:** The cooling and warming rates in the KVS and the control device calculated using a thermal transfer-based simulation method

Device	Cooling rate (°C/min)	Warming rate (°C/min)
KVS	683,000	612,000
Control device	26,000	25,000

*KVS* Kitasato Vitrification System

## Discussion

In the present study, it was demonstrated for the first time that mouse embryos can be successfully vitrified using the KVS. To date, many cryodevices for vitrification, such as the Cryoloop [3], nylon loop [7], open pulled straws [2], the Cryotop [5], and gel-loading tips [6], have been developed. The basic concept of these devices for vitrification is to minimize the volume of the vitrification solution around the embryos to increase the rates of cooling and warming during the vitrification and devitrification processes. In order to address this issue, we previously reported the effectiveness of the KVS as a simple method that vitrifies embryos or oocytes on a membrane filter, which absorbs extracellular vitrification solution [9, 15, 16]. However, because the total light transmittance of the membrane filter is low, it is difficult to observe the embryo deposited on a membrane filter under a stereomicroscope. Therefore, it is almost impossible to check the deposition of the embryo in a reliable manner. In contrast, a notable feature of the KVS in this study is that after the embryo and vitrification solution are placed on the absorber of the device, the embryo can easily be observed under a stereomicroscope (Fig. 2). In the cryopreservation of human embryos, an operator has to observe the embryos on a cryodevice under a stereomicroscope at the time of vitrification. Another feature of the KVS in this study was that the process for absorbing excess vitrification solution around the embryo is simple and stable. In addition, it is easy to control the volume of vitrification solution in the KVS. Therefore, we believe that the KVS is applicable to the cryopreservation of human embryos in ART.

The most notable feature of vitrification using the KVS is the absorption of excess vitrification solution around the embryos to achieve more rapid cooling and warming rates. In the first experiment, we examined the effect of absorbing excess vitrification solution around the embryos to confirm whether this feature of the KVS is effective for the viability of vitrified embryos. As a result, although a high survival rate (100%) was achieved with or without the absorption of excess vitrification solution, the subsequent development rate of embryos, such as the re-expand and hatching rates, were higher than those of embryos in which the excess vitrification solution was not absorbed (Table 2). It must be noted that blastocyst hatching is a manifestation of good-quality embryos and high developmental competence [17]. A possible explanation for the high viability and developmental competence of vitrified embryos achieved using the KVS was that the more rapid cooling and warming rates achieved by absorbing the excess vitrification solution did not result in cryodamage to the embryo during the vitrification and devitrification processes. In fact, in Experiment 4, we confirmed the cooling and warming rates of cells vitrified using the KVS device using a simulation analysis (Figs. 3 and 4).

To date, it is known that the cryotolerance of embryos depends on the developmental stage in many mammalian species. Dhali et al. [18] reported that the modified droplet vitrification (MDV) procedure resulted in better survival at the morula stage than that achieved for zygotes and 2-cell-stage mouse embryos. Accordingly, in Experiment 2, we compared the viability of mouse embryos at the 2-cell stage vitrified or not vitrified. As shown in Table 3, there were no significant differences in the survival or blastocyst formation rates between vitrified or not vitrified embryos. Zhang et al. [19] reported that the survival and blastocyst formation rates of vitrified-warmed mouse embryos vitrified using the Cryotop at the 2-cell stage were 96.0 and 69.4%, respectively. Furthermore, An et al. [20] reported that the blastocyst formation rate (85.7%) of vitrified-warmed mouse embryos using the MDV method at the 2-cell stage was significantly lower than that of non-vitrified embryos. In contrast, the blastocyst formation rate for the KVS in this study was higher than that measured in the previous studies [19, 20] of vitrified mouse embryos. Our results indicate that vitrification using the KVS is effective for the cryopreservation of mouse 2-cell-stage embryos as well as blastocysts.

As shown in Table 4, this is the first report of the ability of mouse embryos, vitrified using the KVS, to develop into live offspring. An et al. [20] reported that when mouse embryos at the 4-cell stage were vitrified using several vitrification methods (e.g., MDV, Cryoloop, and Cryotech) and transferred into recipients, the birth rates ranged between 35.4 and 43.8%, with no significant differences between these rates and those for fresh embryos (45.1%). Similarly, in this study, the rates of development to live offspring for vitrified mouse embryos were not different from those of non-vitrified embryos. These results prove the effectiveness of the KVS.

In Experiment 4, we assessed the cooling and warming rates in the KVS and the control device via a thermal transfer-based simulation method using numerical modeling. This simulation method was previously reported by Tarakanov et al. [14] in a manner similar to this analysis. Indirect methods to estimate the cooling and warming rates of vitrification devices are important tools because it is difficult for direct measuring methods to assess real phenomena in closed spaces, such as closed-type vitrification devices, or small spaces, such as nano-order droplets, as described in this work. On the contrary, Mazur and Seki [21] reported direct measurement methods using thermoelectric coupling and the Cryotop, with a vitrification solution volume of 100 nL and cooling and warming rates of 69,000 and 118,000 °C/min, respectively. The Cryotop, consisting of a polypropylene film with a width and thickness of 0.7 and 0.1 mm, respectively [22], is currently said to be one of the most excellent devices for enabling rapid freezing. To the best of our knowledge, the embryologists used the vitrification solution volume by applying the Cryotop that was within the specified ranges, although it differs greatly between individuals. The cooling and warming rates of the Cryotop device calculated by the indirect measurement method in this study, using previous report parameters, obtained almost the same results as those reported from the direct measurement method by Mazur and Seki [21] (data not shown). In this analysis, the cooling and warming rates of the KVS device, which can be automatically achieved to 1.3 nL, were estimated at 683,000 and 612,000 °C/min, respectively. These rates are extremely high compared to those reported for other vitrification devices [14, 21, 23]. The results of our simulation analysis indicate that decreasing the volume of the vitrification solution around the embryo is critical for increasing the cooling and warming rates. This may be because of the low thermal conductivity of the vitrification solution surrounding the embryo. The thermal distribution using the KVS during the vitrification procedure indicates that thermal transfer to the embryo may exert a dominant influence via a direct pathway from LN_2_ through the vitrification solution surrounding the embryo, as opposed to an indirect pathway through the support under the embryo. Thus, the KVS, which minimizes the vitrification solution surrounding the embryo, may prove to be the preferred device for achieving rapid cooling and warming rates.

## Conclusions

In conclusion, our study clearly demonstrated that the KVS is a novel vitrification device for the cryopreservation of mouse embryos at the blastocyst and 2-cell stage. The KVS should be applicable as a new cryodevice for the cryopreservation of human embryos. However, further study is required to confirm the effectiveness of the device for this purpose.

## Abbreviations

ART: Assisted reproductive technology
KVS: Kitasato vitrification system
